# Youth working in tobacco farming: effects on smoking behavior and association with health status

**DOI:** 10.1186/s12889-020-8169-z

**Published:** 2020-01-20

**Authors:** Ethel Alderete, Jennifer Livaudais-Toman, Celia Kaplan, Steven E. Gregorich, Raúl Mejía, Eliseo J. Pérez-Stable

**Affiliations:** 1 Instituto de Ciencia y Tecnología Regional (ICTER), UE CISOR Consejo Nacional de Investigaciones Científicas y Técnicas/Universidad Nacional de Jujuy, San Salvador de Jujuy, Argentina; 2 Division of General Internal Medicine, Department of Medicine, University of California, San Francisco, USA; 3 Centro de Estudios de Estado y Sociedad (CEDES), Buenos Aires, Argentina; 4 Division of Intramural Research, National Heart, Lung and Blood Institute, and Office of the Director, National Institute on Minority Health and Health Disparities, National Institutes of Health, 6707 Democracy Boulevard, Suite 800, Bethesda, MD 20892 USA

**Keywords:** Tobacco farming, Youth, Smoking, Health Status, Interagency Policy Making

## Abstract

**Background:**

Cultivation of tobacco raises concerns about detrimental health and social consequences for youth, but tobacco producing countries only highlight economic benefits. We compared sociodemographic and health-related characteristics of school-age youth who worked and did not work in tobacco farming and assessed the effects on smoking behavior and health at 1 year.

**Methods:**

We used existing data collected in the province of Jujuy, Argentina where 3188 youth 13 to 17 years of age from a random middle school sample responded to longitudinal questionnaires in 2005 and 2006. Multivariate logistic regression models predicted association of tobacco farming work with health status and smoking behavior at 1 year.

**Results:**

22.8% of youth in the tobacco growing areas of the province were involved in tobacco farming. The mean age of initiation to tobacco farming was 12.6 years. Youth working in farming had higher rates of fair or poor versus good or excellent self-perceived health (30.3% vs. 19.0%), having a serious injury (48.5% vs. 38.5%), being injured accidentally by someone else (7.5% vs. 4.6%), being assaulted (5.5% vs. 2.6%), and being poisoned by exposure to chemicals (2.5% vs. 0.7%). Youth working in tobacco farming also had higher prevalence of ever (67.9% vs. 55.2%), current (48.0% vs. 32.6%) and established smoking (17.8% vs. 9.9%). In multivariate logistic regression models tobacco farming in 2005 was associated with significant increased reporting of serious injury (OR = 1.4; 95%CI 1.1–2.0), accidental injury by someone else (OR = 1.5; 95% 1.0–2.1), assault (OR = 2.2; 95% CI 1.3–3.8), and poisoning by exposure to chemicals (OR = 2.5; 95% CI 1.2–5.4). Tobacco farming in 2005 predicted established smoking 1 year later (OR = 1.5; 95% CI 1.1–2.0).

**Conclusion:**

Youth who work in tobacco faming face a challenging burden of adversities that increase their vulnerability. Risk assessments should guide public policies to protect underage youth working in tobacco farming. (298 words).

## Background

Child labor is regarded as the employment of children less than 18 years of age [1]. It is associated with poverty, inadequate educational opportunities, gender inequality, and a variety of health risks as many are involved in hazardous occupations [2–5]. Children who work have higher rates of mortality, malnutrition and disability compared with those who do not work [6]. An estimated 6 million work-related injuries occur among children that result in 2.5 million becoming disabled and 32,000 fatalities each year [7]. Working children are more susceptible to harm from exposures than adults [8, 9] and more susceptible to emotional and physical abuse and drug addictions [10, 11].

Widespread cultivation of tobacco leaf has raised diverse public health issues including concern for child labor and for occupational health hazards. Children contribute significantly to the tobacco farming workforce in low and middle income countries [12]. In this occupation they are exposed to unsuitable working conditions and toxic chemicals [13]. Pesticides can cause skin and eye irritation, nerve damage, and respiratory symptoms. Dermal absorption of nicotine from contact with wet tobacco leaves can cause green tobacco sickness [14, 15]. Other health effects associated with tobacco farming include, respiratory disorders, musculoskeletal injuries and psychiatric disorders [16–20].

Van Minh et al. (2009) [21] conducted a survey among tobacco and non-tobacco farmers in Vietnam. The occurrence of 9 out of the 16 health problems was higher among tobacco farmers. Tobacco farming was the second predictor of self-reported health problems after the effect of age, placing these workers at increased risk of injury and illness. Similarly, Le Cai (2012) [22] conducted a cross-sectional survey among 8681 adults aged ≥18 years in rural areas of the Yunnan Province, China from 2010 to 2011. Tobacco farmers had higher rates of current smoking, nicotine dependence, and second-hand smoke exposure compared with farmers not engaged in tobacco farming. Most tobacco users (84.5%) reported initiating smoking during adolescence.

In the past 20 years, the tobacco production in Argentina has grown and the country is among the top six worldwide. In 2009/2010 the production reached 132,869 tons, with 37.2% produced in the province of Jujuy. However, the tobacco farming labor force represents highly vulnerable sectors of the population facing poor living and working conditions [23]. More than 50% of the total production is exported in the form of tobacco leaf. In May 2003, Argentina signed the Framework Convention on Tobacco Control, but the agreement has still not been ratified by the National Congress. Nevertheless, in 2007 the National Program for Tobacco Control was established within the Ministry of Health. Several national and local tobacco control laws have been enacted, including the ban on selling tobacco to minors, the regulation of tobacco product advertising and promotion, and indoor tobacco consumption in public places. Regarding child labor, the country has ratified ILO Convention 138 Concerning Minimum Age for Admission to Employment. The Law 26,390 raised from 14 to 16 years the legal age for employment and set special protection for employees between 16 and 18 years of age [24, 25].

There is currently a gap in the state of the knowledge regarding the relationship between tobacco farming and smoking among underage youth in Latin America. This study evaluates the role of working in tobacco farming on tobacco use behavior among underage youth attending schools in the province of Jujuy. The research questions addressed in this analysis were the following: 1. Are the sociodemographic characteristics of youth who work in tobacco farming different from peers not working in tobacco farming? 2. Are indicators of self-reported health status worse among youth working in tobacco farming? 3. Is there an effect of working in tobacco on smoking behaviors at 1 year of follow-up?

## Methods

### Setting

The Province of Jujuy, Argentina is characterized by a geographic configuration that includes lowlands where tobacco farms are located. Tobacco farming is an important contributor to the economy of the province, with 120 to 130 workdays by farmed hectare. The majority of the tobacco workforce in Jujuy are individuals hired by mid to large scale farmers. Only 1% are small farms with less than 2 ha of land that depend solely on family labor [26, 27].

### Sampling

Secondary schools were randomly sampled from within the three geographic areas of Jujuy. Secondary schools include 8th through 12th grades and reflect the standard educational organization in Argentina. Based upon government data, we selected a representative sample of schools containing approximately 1000 eighth grade students from within each geographic area (i.e., disproportionate stratification). The final sample included 27 schools, three of which were private. The baseline data was collected in 2004 (*N* = 4276) among all enrolled 8th grade students, and three follow up surveys were conducted between 2005 and 2007. The response rate for each follow up was 94.2, 91.7 and 80.0% respectively. Surveys were self-administered in class with research staff and school coordinators present as proctors. In each school, one attempt was made to survey absent students at a subsequent date. The detailed study procedures have been described in a previous publication [28]. For this report we used data from the 3234 students between ages 13 and 17 years who completed surveys in 2005 (T1) and 2006 (T2). Of these, 46 (1.4%) did not answer the questions about tobacco farming, yielding a total sample of 3188. The UCSF Committee on Human Research and an NIH-certified human subjects research board in Buenos Aires based at Centro de Educación Médica e Investigaciones Clínicas (CEMIC) approved the research protocol. Passive consent was requested from caretakers and students signed an active consent.

### Questionnaire development

The questionnaire consisted of translated items from surveys of adolescents in the U.S. [29], and questions developed through qualitative research in the target population [28]. Items in English were translated and reviewed by three Argentinean investigators and two other Spanish-speaking research staff. Pilot testing of the instrument was conducted with students in rural and urban areas evaluating situational factors, content, characteristics of the respondents, and time of administration that averaged 1 h.

### Demographics

Sociodemographic variables were extracted from baseline data including sex, age, ethnicity (Indigenous, mixed Indigenous and European, European), and religion. Religion was categorized as Catholic, Christian or Evangelical, and others corresponding to low frequency religions. A binary (yes/no) low socioeconomic status (SES) variable was developed by classifying the primary caretaker as having up to primary education, being unemployed, or being on welfare, versus having a higher education level or being formally employed. The location of the school was reported in the questionnaire by interviewers.

### Health related factors

Health related variables correspond to T1 responses. Respondents provided a self-assessment of their health status, categorized as excellent, good, fair or poor. Another set of questions probed on the occurrence of injuries. We asked if in the previous year respondents had a serious injury, if they were injured accidentally by someone else, if they had been assaulted, and if they had been poisoned by exposure to chemical products. Local agricultural workers commonly refer to pesticides as “chemicals” and the survey question was phrased accordingly.

### Smoking behavior

For this study, we used smoking information from T1 (2005) and T2 (2006). Smoking behavior was the main outcome and questions were developed to be comparable to those used in the Centers for Disease Control and Prevention GYTS survey [29]. Respondents were considered ever smokers if they tried at least a cigarette puff in their lifetime and never smokers had not tried even one puff. Current smokers were defined as having smoked at least one whole cigarette in their lifetime and at least one puff in the previous 30 days. Established smokers were defined as current smokers who had smoked at least 100 cigarettes in their lifetime. Respondents also reported on the number of friends who smoked (none, 1 to 4, 5 or more), and whether any adult smoked in their home.

### Working in tobacco farming

Hereby reported exposure variables correspond to measurements at T1. The youth were asked if they had ever worked in any of the tasks involved in tobacco production, growing, harvesting or selecting tobacco leaf, without discriminating the different types of tasks. Youth reported their age of initiation in tobacco farming work. Information about working in non-tobacco farming occupations was also requested.

### Data analysis

The sampling design was incorporated into all models by specifying geographic areas as strata and schools as clusters as well as including weights to adjust for disproportionate stratification. In addition, a finite population correction was applied to adjust for the relatively large proportion of available schools sampled within each geographic area. The statistical program Stata (version 14.2) was used for data analysis. Standard errors and confidence intervals were estimated via the Taylor expansion approximation using the *svy* procedures in Stata [30]. First, we conducted descriptive analyses by sex, to profile the sample. We calculated the prevalence of ever, current and established smoking, with chi square tests and *p* values at T1 and T2, and the percentage of youth who reported at T1 that they had ever worked in tobacco and non- tobacco farming. The mean and standard deviation of the age for girls and boys, and of the age of initiation in tobacco farming was calculated. Bivariate contingency tables examined the pairwise relationship of sociodemographic characteristics, health related factors and smoking behavior by sex, and by working in tobacco farming. Bivariate analysis also examined the pairwise relationship of non-tobacco farming and the smoking behavior variables.

Multivariate logistic models regressed working in tobacco farming at T1 with each of the health-related variables at T1. Separate multivariate logistic models regressed working in tobacco farming at T1 onto cigarette smoking behaviors at T2 (ever, current or established smoking). Covariates included sociodemographic characteristics (sex, age, low SES, ethnicity, religion, number of friends who smoked, adult smokers at home, and for each model, the corresponding smoking behavior at T1 (ever, current or established smoking). We estimated adjusted odds ratios and 95% confidence intervals.

## Results

The mean age for girls was 14.5 years (95% CI 14.4–14.6) and for boys, 15.1 years (95% CI 14.9–15.3) (data not shown). Low SES was more prevalent among girls than boys (25.8% vs. 20%, *p* = 0.006) and 71.7% of the girls self-identified as being Indigenous, compared with 65% of the boys (*p* < 0.001). A greater percentage of girls reported living with an adult who smoked at home (76.8% vs. 73.2%, *p* = 0.018).

A greater percentage of girls perceived that their health status was fair or poor, compared with boys (25.3% vs. 14.3%, *p* < 0.001). However, boys were more likely to report serious injures (46.9% vs. 33.5%, *p* < 0.001), being accidentally injured by someone else (6.2% vs. 4.0%, *p* = 0.002) and being assaulted (4.5% vs. 1.6%, *p* < 0.001) (Table 1). At T1 (2005), the prevalence of ever (56.6%) and current smoking (34.4%) was similar for boys and girls but established smoking was more prevalent among boys (13% vs. 8.9%, *p* = 0.004). The prevalence of working in tobacco farming was unevenly distributed across geographical regions, involving 22.8% of youth in the lowlands where tobacco is cultivated, and between 4.5 to 4.9% in the other areas (data not shown). Ever working in tobacco farming was reported by 11.5% of the total sample (Table 1). Involvement in tobacco farming was more prevalent among boys (12.9% vs. 10.3%, *p* = 0.044) but the mean age of initiation did not differ significantly between girls (12.0; 95% CI 11.4–13.0) and boys (12.7; 95% CI 12.1–13.2).

**Table 1:** Sociodemographic characteristics of 3188 Youth by sex, Jujuy, Argentina, 2005I

	Girls N (%) *N* = 1739	Boys N (%) *N* = 1495	Total N (%) *N* = 3234	*p* value
Sociodemographic characteristics				
*Low SES*	457 (25.8)	314 (20.0)	771 (23.1)	0.006*
*Ethnicity*				
Indigenous	1215 (71.7)	975 (65.0)	2190 (68.6)	0.001*
Mixed Indigenous/European	342 (21.5)	307 (22.8)	649 (22.1)	
European	101 (6.8)	150 (12.1)	251 (9.3)	
*Religion*				
Catholic	1461 (85.2)	1248 (85.1)	2709 (85.1)	0.887
Evangelical	186 (10.8)	159 (10.4)	345 (10.6)	
Other	67 (4.0)	67 (4.5)	134 (4.2)	
*Number of friends who smoke*				
None	454 (27.6)	320 (23.4)	774 (25.7)	0.056
1–4	446 (27.1)	345 (24.8)	791 (26.1)	
5+	720 (45.3)	724 (51.8)	1444 (48.3)	
*Adult smokers at home*	1314 (76.8)	1076 (73.2)	2390 (75.2)	0.018*
Health variables				
*Perceived health*				
Excellent/good	1232 (74.7)	1240 (85.7)	2472 (79.7)	0.001*
Fair/poor	479 (25.3)	225 (14.3)	704 (20.3)	
*Serious injury*	590 (33.5)	678 (46.9)	1268 (39.6)	0.001*
*Accidental injury by someone else*	67 (4.0)	97 (6.2)	164 (5.0)	0.002*
*Assaulted*	28 (1.6)	64 (4.5)	92 (2.9)	0.001*
*Poisoned by chemical products*	11 (0.8)	17 (1.0)	28 (0.9)	0.452
Smoking Prevalence T1 (2005)				
*Ever smoker*	969 (57.0)	841 (56.2)	1810 (56.6)	0.691
*Current smoker*	561 (33.7)	521 (35.1)	1082 (34.4)	0.7181
*Established smoker*	146 (8.9)	194 (13.0)	340 (10.8)	0.004*
Tobacco farming				
*Ever worked in Tobacco Farming*	190 (10.3)	193 (12.9)	383 (11.5)	0.044*
Age started working in tobacco farming, Mean years (95% CI)	12.0 (11.4–13.0)	12.7 (12.1–13.2)	12.2 (11.8–12.6)	

* Chi square test, *p* value

### Tobacco farming sociodemographic, health factors and smoking behavior

The percentage of youth who endorsed an evangelical religion was greater among those who worked in tobacco farming (17.0% vs. 9.8%). Working in tobacco farming was also associated with having low SES (28.5% vs. 22.4%), being Indigenous (77.8% vs. 67.4%), and having more than 5 friends who smoked (60.6% vs. 46.7%).

Among youth working in tobacco farming 30.3% reported perceiving that their health was fair or poor, compared with 19.0% of other youth (*p* = 0.004). Tobacco farming was also associated with having a serious injury (48.5% vs. 38.5%, *p* = 0.004), being injured accidentally by someone else (7.5% vs. 4.6%, *p* = 0.01), being assaulted (5.5% vs. 2.6%, *p* = 0.008), and being poisoned by chemical products (2.5% vs. 0.7%, *p* = 0.003).

Youth who had ever worked in tobacco farming had significantly higher prevalence of ever smoking (67.9% vs. 55.2%, *p* < 0.001), current smoking (48.0% vs. 32.6%, p < 0.001) and established smoking (17.8% vs. 9.9%, *p* = 0.002) at T1 (2005). The prevalence of smoking behaviors increased slightly at T2 (2006) for the total sample. Although smoking rates decreased slightly among youth working in tobacco farming from T1 to T2, they remained significantly higher compared to those who did not work (Table 2). Smoking rates did not differ significantly between youth working any non-tobacco farming job compared to those not working at all for ever smoking (64% vs. 58%, *p* = 0.118), for current smoking (39% vs. 35%, *p* = 0.087) or for established smoking (14% vs. 11%, *p* = 0.189) (data not shown).

**Table 2:** Sociodemographic and health factors associated with tobacco farming in 3188 Youth, Jujuy, Argentina, 2005–2006

	Tobacco Farming			
	No N (%) *N* = 2805	Yes N (%) *N* = 383	Total N (%) *N* = 3188	Chi square *p* value
Sociodemographic characteristics				
*Sex*				0.044
Girls	1524 (55.0)	190 (48.7)	1714 (54.3)	
Boys	1281 (45.0)	193 (51.3)	1474 (45.7)	
*Religion*				0.003
Catholic	2408 (86.0)	301 (78.3)	2709 (85.1)	
Evangelical	280 (9.8)	65 (17.0)	345 (10.6)	
Other	117 (4.2)	17 (4.7)	134 (4.2)	
*Low SES*	660 (22.4)	111 (28.5)	771 (23.1)	0.023
*Ethnicity*				0.034
Indigenous	1899 (67.4)	291 (77.8)	2190 (68.6)	
Mixed Indigenous/European	582 (22.6)	67 (18.3)	649 (22.1)	
European	237 (9.9)	14 (3.9)	251 (9.3)	
*Number of friends who smoke*				0.001
None	711 (26.7)	63 (17.6)	774 (25.7)	
1–4	710 (26.6)	81 (22.0)	791 (26.1)	
5+	1231 (46.7)	213 (60.4)	1444 (48.3)	
*Adult smokers at home*	2102 (75.2)	288 (74.6)	2390 (75.2)	0.089
Health variables				
*Perceived health*				0.004
Excellent	1247 (47.4)	111 (30.9)	1358 (45.5)	
Good	964 (33.6)	150 (38.8)	1114 (34.2)	
fair/poor	586 (19.0)	118 (30.3)	704 (20.3)	
*Had a serious injury*	1092 (38.5)	176 (48.5)	1268 (39.6)	0.004
*Injured accidentally by someone else*	137 (4.6)	27 (7.5)	164 (5.0)	0.010
*Assaulted*	75 (2.6)	17 (5.5)	92 (2.9)	0.008
*Poisoned by chemical products*	8 (0.7)	0 (2.5)	28 (0.9)	0.003
Smoking Behavior T1 (2005)				
*Ever smoker*	1553 (55.2)	257 (67.9)	1810 (56.6)	0.001
*Current smoker*	905 (32.6)	177 (48.0)	1082 (34.4)	0.001
*Established smoker*	276 (9.9)	64 (17.8)	340 (10.8)	0.002
Smoking Behavior T2 (2006)				
*Ever smoker*	1516 (58.9)	237 (68.8)	1753 (60.0)	0.001
*Current smoker*	855 (34.0)	153 (44.9)	1008 (35.2)	0.001
*Established smoker*	276 (11.1)	58 (17.5)	334 (11.9)	0.002

### Effects of exposure to tobacco farming: multivariate analysis

In multivariate logistic regression models working in tobacco farming in 2005 significantly increased the likelihood of having a serious injury, being injured accidentally by someone else, being assaulted or being poisoned by chemical products in the same year (Table 3). In another set of multivariate logistic regression models, tobacco farming in 2005 predicted established smoking 1 year later (2006) (OR = 1.5; 95% CI 1.1.-2.0) (Table 4). Tobacco farming in 2005 was not predictive of ever or current smoking in 2006 although the point estimate was in the increased odds direction. Significant risk factors for established smoking were, religion other than catholic or evangelical, mixed Indigenous-European ethnicity, and having 5 or more friends who smoked versus none (data not shown). Separate logistic models including interaction terms between tobacco farming and sex, ethnicity, and having friends who smoked, yielded no significant interaction effects (data not shown).

**Table 3:** Exposure to Tobacco Farming associated with Health Outcomes Jujuy, Argentina, 2005

Logistic Regression of Exposure to Tobacco Farming in 2005 and Health Outcomes in 2005, for Health-related Outcomes					
	Perceived Health Status Excellent/Good vs. Fair/poor OR (95% CI)	Serious Injury Yes vs. No OR (95% CI)	Accidental Injury by Someone Else Yes vs. No OR (95% CI)	Assault Yes vs. No OR (95% CI)	Chemical poisoning Yes vs. No OR (95% CI)
Tobacco Farming	0.7 (0.5–1.0)	1.4 (1.1–2.0)*	1.5 (1.0–2.1)*	2.2 (1.3–3.8)**	2.5 (1.2–5.4)**

Logistic regression models (3.a. and 3.b.) controlling for age, sex, ethnicity, religion, low SES status, number of friends who smoke, adult smokers at home, and smoking at T1

*p < 0.05; **p < 0.01

**Table 4:** Exposure to Tobacco Farming in 2005 Effects on Smoking Behavior in 2006, Jujuy, Argentina, 2005–2006

Logistic Regression of Exposure to Tobacco Farming in 2005 as a Predictor of Smoking Behaviors in 2006					
	Ever Smoker Yes vs. No OR (95% CI)	Current Smoker Yes vs. No OR (95% CI)	Established smoker Yes vs. No OR (95% CI)		
Tobacco Farming	1.2 (0.9–1.7)	1.2 (0.8–1.6)	1.5 (1.1–2.0)**		

Logistic regression models (3.a. and 3.b.) controlling for age, sex, ethnicity, religion, low SES status, number of friends who smoke, adult smokers at home, and smoking at T1

*p < 0.05; **p < 0.01

## Discussion

The sociodemographic profile of these Jujuy youth working in tobacco farming highlights the roots of the child labor problem at a global level, involving youth belonging to poor families and of non-dominant social groups, particularly Indigenous populations. Our results highlight that socioeconomically vulnerable youths may be further impaired in their development by occupational health problems and the increased risk of cigarette smoking associated with a large set of health risks throughout the life course [13]. Furthermore, youth who worked in tobacco farming reported having a fair or poor self-reported health status in a greater proportion than other youth, as well as increased rates of exposure to toxic chemicals. Although we cannot ascertain the precise nature of sustained injuries, or a direct relation to the occupational context, we identified an increased risk of exposure to violence through assaults among youth working in tobacco farming. The increased exposure to interpersonal violence finding has not been reported in other studies, largely of adult populations. As a primary finding we ascertained a one-year effect of work in tobacco farming among youth, on being an established smoker defined as current smoker of at least 100 cigarettes lifetime. To our knowledge, this finding has not been previously reported and is unique in focusing on underage youth.

Prior research postulates that there may be an association between exposure to pesticides and mental health problems [31–35], and smoking has been associated to psychological distress among adolescents [36]. On this basis, future research could investigate the mediating role of mental health status on smoking among youth who work in tobacco farming. In addition, violence, and its associated stress, may trigger increased desire to smoke linked to coping mechanisms [37]. Not only does working in tobacco farming increase the risk of established smoking, but tobacco related illnesses caused by smoking may be compounded by the occupational hazards of tobacco farming. For example, respiratory illnesses caused by particles and microorganisms growing on tobacco leaves may exacerbate damage to lung cells [19, 18]. Future studies should evaluate synergistic adverse health effects between smoking and occupational hazards in tobacco farming.

This study’s strengths include the population-based sampling strategy which enhances the generalization of results. The repeated sampling of participants between 2005 and 2006 was useful for examining longitudinal effects of tobacco farming. However, with this dataset we were not able to determine a precise date of initiation in tobacco farming to calculate a time of exposure variable, as this information is based on recall and it is not unusual for children to become involved in this activity at very early ages. Likewise, we were not able to determine the type of tasks performed and the amount of time in months and years of previous exposure or if respondents were still working in tobacco farming at the time of the study. The inclusion of youth with less exposure time to tobacco farming work would potentially reduce the effect of the exposure and bias results towards the null. Therefore, we are presenting conservative results. Although the one-year time frame used for this analysis is a limitation and was based on the fact that tobacco farming work questions were not consistently included in all waves of the study. In addition, we cannot ascertain that injuries and poisoning with chemicals occurred while conducting tobacco farming activities. Another limitation is that we are not able to draw causal inference about health-related factors since the health data used for this analysis was collected at only one time point. Although the data were collected more than 10 years ago, the practice of hiring underage youth in tobacco farming is a current practice [38].

More than 250,000 hectares of tobacco are planted throughout the globe in more than a 100 countries [39]. However, the governments of tobacco producing countries largely view tobacco cultivation as an important contributor to the national economy by generating tax revenues, employment and income in otherwise deprived areas, while overlooking labor rights and health issues, including the economic costs of illness and social problems related to tobacco farming [38].

This report contributes to breaching the knowledge gap of the longitudinal effect of tobacco farming on smoking behavior among youth. In addition, poor self-perceived health, more accidents and exposure to violent settings in this population, highlights the need to develop structural mechanisms to protect youth from the often overlooked social and health risks involved in tobacco farming. In the process of setting national economic priorities and policies, state agencies other than those pertaining to the economic sector, namely health, social work, education, environmental and other related agencies, practitioners and scholars should be called upon to contribute to diagnostic assessments and policy formulations that take into account the complex nature of tobacco farming.

## Conclusions

Tobacco farming work by underage youth in the Province of Jujuy, Argentina is associated with adverse health events, worse perceived health status and greater odds of becoming established smokers. Risk assessments should guide public policies to protect underage youth working in tobacco farming through structural change and enforcement of existing regulations.

## Data Availability

The datasets used and/or analyzed during the current study are available from the corresponding author upon request.

## Abbreviations

CEMIC: Centro de Educación Médica e Investigaciones Clínicas
OR: Odds Ratio
T1: Time 1
T2: Time 2
UCSF: University of California, San Francisco
SES: Socioeconomic status
